# Zephycandidine A, the First Naturally Occurring Imidazo[1,2-*f*]phenanthridine Alkaloid from *Zephyranthes candida*, Exhibits Significant Anti-tumor and Anti-acetylcholinesterase Activities

**DOI:** 10.1038/srep33990

**Published:** 2016-09-23

**Authors:** Guanqun Zhan, Xiaolan Qu, Junjun Liu, Qingyi Tong, Junfei Zhou, Bin Sun, Guangmin Yao

**Affiliations:** 1 Hubei Key Laboratory of Natural Medicinal Chemistry and Resource Evaluation, School of Pharmacy, Tongji Medical College, Huazhong University of Science and Technology, Wuhan 430030, People’s Republic of China; 2 School of Pharmaceutical Sciences, Taishan Medical University, Tai-An 271016, People’s Republic of China

## Abstract

Zephycandidine A (1), the first naturally occurring imidazo[1,2-*f*]phenanthridine alkaloid, was isolated from *Zephyranthes candida* (Amaryllidaceae). The structure of 1 was elucidated by spectroscopic analyses and NMR calculation, and a plausible biogenetic pathway for zephycandidine A (1) was proposed. Zephycandidine A (1) exhibited significant cytotoxicity against five cancer cell lines with IC_50_ values ranging from 1.98 to 7.03 *μ*M with selectivity indices as high as 10 when compared to the normal Beas-2B cell. Further studies suggested that zephycandidine A (1) induces apoptosis in leukemia cells by the activation of caspase-3, upregulation of Bax, downregulation of Bcl-2, and degradation of PARP expression. In addition, zephycandidine A (1) showed acetylcholinesterase (AChE) inhibitory activity, and the docking studies of zephycandidine A (1) and galanthamine (2) with AChE revealed that interactions with W286 and Y337 are necessary.

Amaryllidaceae alkaloids are an important resource for new drug discovery, among them, galanthamine, a well-known acetylcholinesterase (AChE) inhibitor, is a first-line treatment of Alzheimer’s disease. More than 500 amaryllidace alkaloids belonging to 18 framework types have been reproted to possess AChE inhibitory, analgesic, antibacterial, antifungal, antimalarial, antitumor, and antiviral activities^1^. *Zephyranthes candida* (Lindl.) Herb., an amaryllidaceous bulbous herb, is widely cultured as an ornamental flower, and the whole plants are used as a folk medicine in China to treat infantile convulsions, epilepsy, and tetanus^2^. In 1990, Pettit group reported the principal cytostatic component, *trans*-dihydronarciclasine, from the extract of *Z. candida* collected in China^3^. In a previous study on the cytotoxic alkaloids of *Z. candida* collected in June 2010 at Shiyan, China, total 15 alkaloids were isolated, and some of them exhibited signficant cytotoxicity against five cancer cell lines^4^. Among them, new alkaloid *N*-methylhemeanthidine chloride showed potent inhibitory activities against pancreatic cancer *in vitro* and *in vivo*, and the mechanism was found to be via down-regulating AKT activation^5^.

In order to search for more active alkaloids from *Z. candida*, the whole plants were re-collected at the same place on October 2011^6^. Further study on this collection led to the isolation of a new minor alkaloid with a rare imidazo[1,2-*f*]phenanthridine framework, which was named zephycandidine A (**1**) (Fig. 1). Phenanthridine alkaloids are very common in the Amaryllidaceae plants^1^, however, there is no report of the presence of the imidazo[1,2-*f*]phenanthridine type alkaloids, although imidazo [1,2-*f*] phenanthridine was synthesized as a synthon by Cronin group in 2006^7^. Zephycandidine A (**1**) represents the first example of a naturally occurring imidazo[1,2-*f*]phenanthridine type alkaloid. In this paper, we describe the isolation, structure determination, plausible biosynthetic pathway, cytotoxicity and inducing apoptosis in leukemia cells, AChE inhibitory activity, and the docking study with AChE of zephycandidine A (**1**).

## Results and Discussion

### Structural elucidation of zephycandidine A (1)

**Figure 2:**
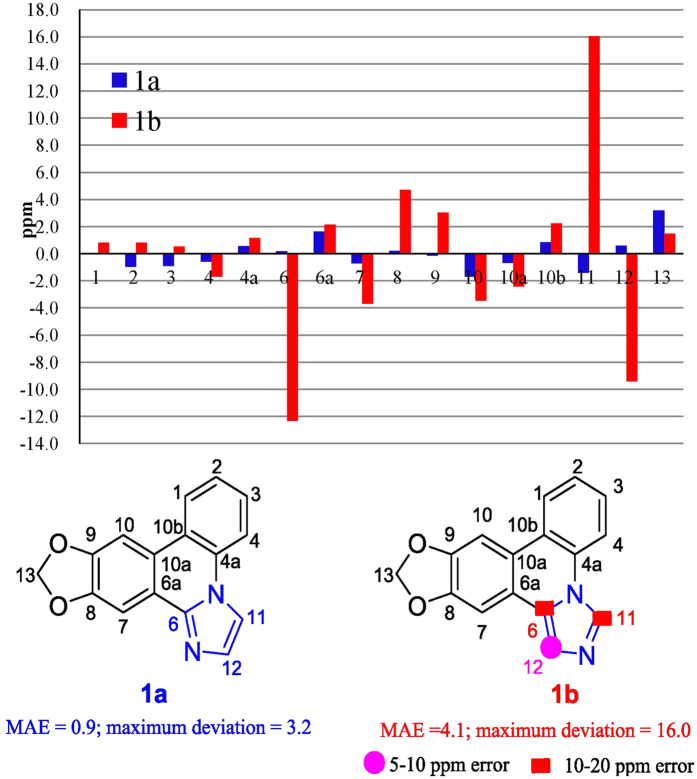
Structures and differences in ppm between calculated and experimental ^13^C NMR shifts for **1a** and **1b**.

Zephycandidine A (**1**) was isolated as colorless oil. The molecular formula C_16_H_10_N_2_O_2_ was determined by the HRESIMS ion at *m/z* 263.0789 [M + H]^+^ (calcd for 263.0821) in conjunction with the ^13^C NMR data, requiring thirteen degrees of unsaturation. The ^1^H and ^13^C NMR data of **1** (Table 1) were very similar to those of trisphaeridine, which was co-isolated from this plant^4^. The major differences between them were that the methine (*δ*_H_ 9.07, s, H-6; *δ*_C_ 152.0, C-6) at C-6 in trisphaeridine is replaced by a quaternary olefinic carbon (*δ*_C_ 143.8, C-6) in **1**, and there are two additional olefinic methines (*δ*_H_ 7.52, d, *J* = 1.4 Hz, H-11; 8.32, d, *J* = 1.4 Hz, H-12; *δ*_C_ 113.8, C-11; 131.5, C-12) in **1**. The trisphaeridine unit accounts for eleven degrees of unsaturation, two additional olefinic methines account for one degree of unsaturation, and the remaining one degree of unsaturation suggested the presence of additional ring in **1**. Due to the small coupling constant *J* = 1.4 Hz between H-11 and H-12, as well as the observed the correlations of H-11 to H-12 in ^1^H-^1^H COSY spectrum, H-11 and H-12 should be in a *meta* or *ortho* position of a five-membered ring. In consideration of the molecular formula and chemical shifts, there are two possible structures **1a** and **1b** (Fig. 2) for **1**. However, there were no obvious HMBC correlations from H-11 and H-12 to C-4a or C-6a. So, it is impossible to verify the structure of **1** by the HMBC analysis.

To determine the structure of **1**, the ^13^C NMR shifts of two possible structures **1a** and **1b** (Table 1) were calculated with the GIAO option using the B3LYP/6-31G* DFT method^8^. According to the method of P. Crews *et al.*^9^, the corrected ^13^C NMR shifts for **1a** and **1b** (Table 1) were calculated, and then, the differences between the experimental and corrected ^13^C NMR shifts for **1a** and **1b** were analysed (Fig. 2). As shown in Fig. 2, the MAE (mean absolute error) and MD (maximum deviation)^9^ for **1a** were 0.9 and 3.2 ppm, respectively, which are acceptable to meet MAE < 2.2 and MD < 5^9^. However, the MAE and MD for **1b** were 4.1 and 16.0 ppm, respectively, which are far higher than standard of MAE < 2.2 and MD < 5. Especially, the absolute error of atoms at C-6, C-11, and C-12 were much higher than 5 ppm. Thus, the chemical structure of **1** should be **1a**, instead of **1b**.

Furthermore, a weak correlation between H-12 and H-11 was finally observed in the NOESY spectrum of **1** when the acquisition time for the NOESY data was increased much more, which further supported the structure of **1a**. Therefore, the structure of zephycandidine A (**1**) was assigned to [1,3]dioxolo[4,5-*j*]imidazo[1,2-*f*]phenanthridine.

### Proposed biosynthetic pathway for zephycandidine A (1)

**Figure 3:**
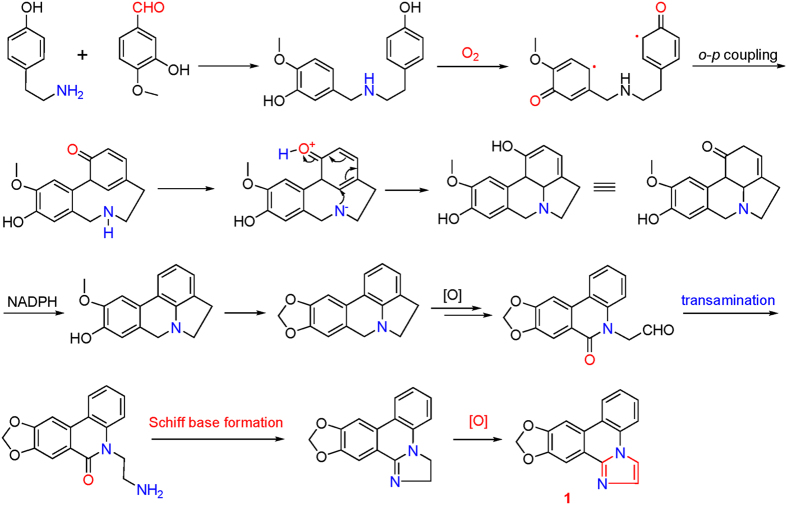
Plausible biogenetic pathway for zephycandidine A (**1**).

Although the imidazo-phenanthridine synthon had been produced in a five-step one pot reaction by Cronin group^7^, the biosynthetic pathway of **1** in *Z. candida* (Fig. 3) could be traced back to tyramine and isovanillin to form a benzylphenethylamine alkaloid, following by the oxidative coupling of phenols to form a lycorine-type alkaloid. After oxidation, transamination, intramolecular Schiff base formation, and final oxidation, the lycorine-type alkaloid intermediate was transformed to be the imidazo[1,2-*f*]phenanthridine alkaloid **1**.

### Cytotoxity of zephycandidine A (1)

**Figure 4:**
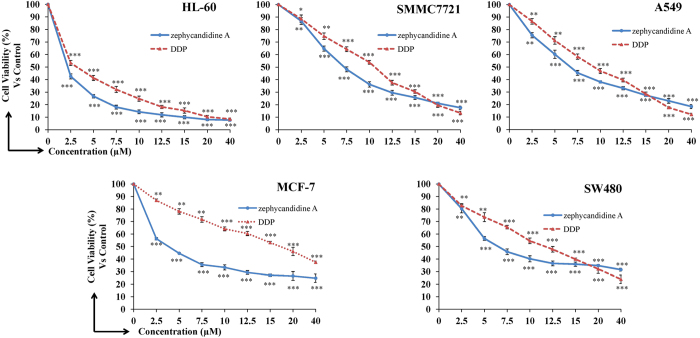
Cytotoxity of zephycandidine A (**1**) against five human cancer cell lines (human myeloid leukemia HL-60, hepatocellular carcinoma SMMC-7721, lung cancer A549, breast cancer MCF-7, and colon cance4r SW480). Cells were treated with various concentrations of zephycandidine A for 48 h and cell viability was determined by MTT assays using DDP as positive control. Data are presented as the means ± SD of three experiments, P values were derived from Student’s *t*-test (*P < 0.05, **P < 0.01, ***P < 0.001), compared to the control group.

Cytotoxity of zephycandidine A (**1**) against five human cancer cell lines, human myeloid leukemia HL-60, lung cancer A549, breast cancer MCF-7, colon cancer SW480, and hepatocellular carcinoma SMMC-7721, together with one non-cancerous cell line human bronchial epithelial cells Beas-2B, were evaluated using the MTT (3-(4,5-dimethylthiazol-2-yl)-2,5-diphenyltetrazolium bromide) method^10^. DDP (*cis*-platin) and DMSO were used as the positive and negative controls, respectively. The results were showed in Fig. 4 and Table 2. Zephycandidine A (**1**) showed more potent cytotoxicity against five human cancer cell lines than the positive control (*cis*-platin) with IC_50_ values of 1.98, 6.49, 3.44, 6.27, and 7.03 *μ*M, respectively. More interestingly, **1** showed weak cytotoxicity against the normal Beas-2B cell line (IC_50_ = 20.08 *μ*M) with the selectivity indices as high as 10.1.

### Morphological changes of HL-60 cells treated with zephycandidine A (1)

**Figure 5:**
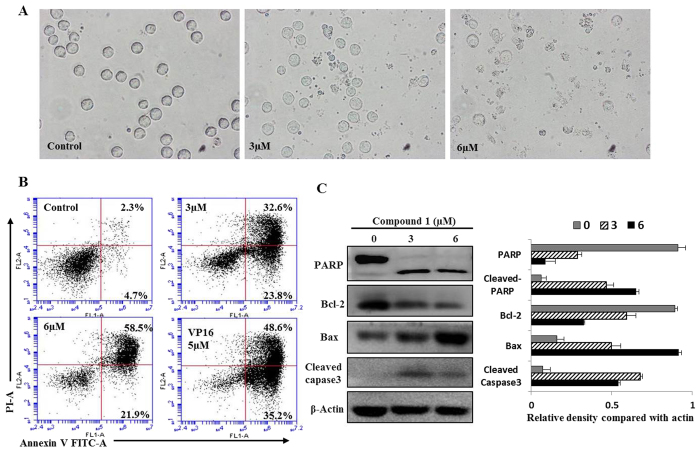
Zephycandidine A (**1**) induces apoptosis in leukemia cells. (**A)** Morphological changes of HL-60 cells after treated with zephycandidine A (**1**) for 48 h. Phase-contrast microscopic view, magnification 400 × . (**B)** Apoptosis was determined by Annexin V-FITC and PI staining using flow cytometric analysis. Cells in the lower right quadrant indicate early apoptotic cells, and cells in the upper right quadrant indicate late apoptotic cells. (**C)** Western blot analysis for PARP, Bcl-2, Bax and caspase-3 levels, *β*-Actin used as a loading control. The relative density of each protein was detected by Image J, data are presented as the means of three experiments, bars, SD.

To further analysis the anticancer effects of zephycandidine A (**1**), we used HL-60 as a model. The morphological changes and cytoclasis of HL-60 cells were examined under an inverted phase contrast microscope. As shown in Fig. 5A, cell shrinkage and abnormal morphological features could be observed in 3.0 and 6.0 *μ*M zephycandidine A (**1**)-treated groups.

### Zephycandidine A (1) causes HL-60 cells death by apoptosis

To ensure whether zephycandidine A (**1**) meditated inhibition of growth and proliferation was associated with apoptosis, the flow cytometry apoptosis assay was performed using Annexin V and PI staining^11^. After treatment at various concentrations (0, 3.0, and 6.0 *μ*M) for 48 h, the apoptosis of the cells were detected. Compared with the untreated cells, the treatment of 6 μM zephycandidine A (**1**) can result by up to 80.4% apoptosis incidence (Fig. 5B). As a positive control, the treatment of VP16 (etoposide phosphate, 5 *μ*M) leads to 83.8% apoptosis incidence (Fig. 5B). These results demonstrate that zephycandidine A (**1**) induced apoptosis of HL-60 cells.

### Zephycandidine A (1) changes the expression of apoptotic-related proteins

As we know, among the caspase-family, caspase-3 is considered to be a critical effector, and the activation of caspase-3 could result in PARP degradation^12^. Bcl-2 family also plays a central regulatory role in the mitochondrial pathway of apoptosis, the balance between Bcl-2 and Bax is critical for cell survival and death^13^.

HL-60 cells were treated with zephycandidine A (**1**) as describe above. Changes of apoptosis-related protein, including Bax, Bcl-2, cleaved caspase-3 and PARP, were assessed by western blot analysis. *β*-actin was used as an internal control, and relative optical density of proteins were compared with *β*-actin. As shown in Fig. 5C, zephycandidine A (**1**) treatment altered the expression levels of PARP and cleaved-caspase 3, it also down regulated Bcl-2 and upregulated Bax. Taken together, these data suggest that zephycandidine A (**1**)-induced apoptosis in leukemia cells is mediated by the activation of caspase-3, upregulation of Bax, downregulation of Bcl-2 and degradation of PARP.

### Zephycandidine A (1) exhibites acetylcholinesterase inhibitory activity

**Figure 6:**
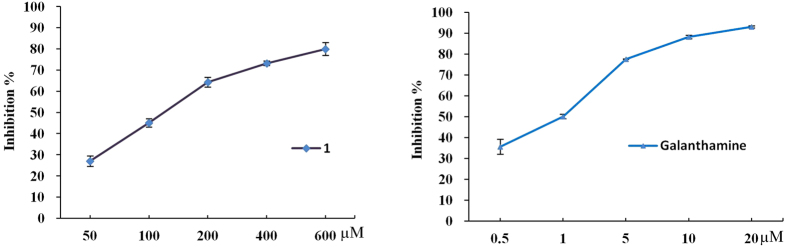
AChE inhibitory activities of zephycandidine A (**1**) and galanthamine (**2**).

Acetylcholinesterase (AChE) activity was measured using a 96-well microplate reader based on Ellman’s method^14^ with slight modification^15^. As showed in Fig. 6, zephycandidine A (**1**) showed AChE inhibitory activity in a dose-dependent manner, and the IC_50_ value was calculated to be 127.99 *μ*M. While, the positive control, galanthamine (**2**), exhibited potent AChE inhibitory activity with an IC_50_ value of 1.02 *μ*M.

### Docking studies on zephycandidine A (1) and galanthamine (2) with AChE

**Figure 7:**
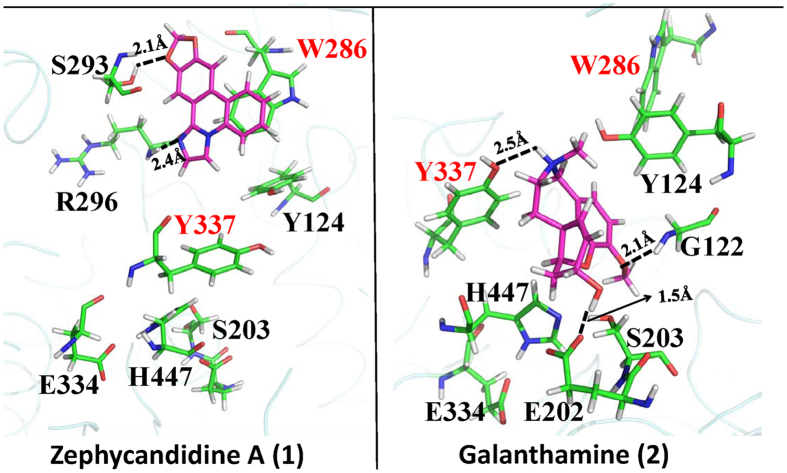
The binding modes of zephycandidine A (**1**) and galanthamine (**2**) in AChE. The ligand atoms are shown in purple stick. The relevant AChE residues are shown in green sticks. The oxygen, nitrogen, and hydrogen atoms are colored in red, blue, and white, respectively.

To study the relationship of the structures and the AChE inhibitory activities, the binding modes of zephycandidine A (**1**) and galanthamine (**2**) with AChE have been obtained by our comprehensive docking studies (Fig. 7). There are two binding sites in the gorge of AChE, i.e., the catalytic binding site in the bottom of gorge and the peripheral anionic site at the lip of the gorge. As shown in Fig. 7, galanthamine (**2**) binds at the catalytic binding site, whereas zephycandidine A (**1**) binds at the peripheral anionic site. Galanthamine (**2**) is stabilized by forming three hydrogen bonds with E202, Y337 and G122, respectively. In the peripheral anionic site, zephycandidine A (**1**) binds the enzyme primarily by the π–π stacking interaction with W286 and by two hydrogen bonds with S293 and R296, respectively.

Extensive studies on the binding of ligands with AChE shows that W286 in peripheral anionic site and Y337 in catalytic binding site play an important role more than stabilizing the binding with the ligands. It has been shown that the loss of interaction with W286 resulted in the loss of binding with AChE, while the ligand is still able to bind butyrylcholinesterase (BuChE)^16^. BuChE has similar but larger gorge than AChE and does not have tryptophan residue in the peripheral anionic site. It is thus suggested that interaction with W286 is the key for ligands to bind AChE. Similarly, Y337 is characterized as “swinging gate” of the catalytic binding site^17^. It hinders the ligands from entering the catalytic binding site, but stabilizes the ligand after it moves into the catalytic site^18^. Recent crystal structures also show there is hydrogen bond between Y337 and quaternary ammonium of ligands^19^. Clearly, W286 and Y337 play an important role in recognizing the ligands. Unable to bind these two residues is very likely unable to enter the peripheral anionic site or the catalytic binding site.

As discussed above, zephycandidine A (**1**) and galanthamine (**2**) interact with the key W286 and Y337 residues, respectively, suggesting that zephycandidine A (**1**) and galanthamine (**2**) do bind AChE. This explains why the inhibition activities against AChE were observed for zephycandidine A (**1**) and galanthamine (**2**). The potential AChE inhibitors are required to interact with W286 to bind at peripheral anionic site, or to interact with both W286 and Y337 to bind at catalytic binding site. This suggested that without obvious interaction with these two residues, no matter how tight binding it shows, the ligand just cannot move into the gorge and therefore express no or few inhibitory activity against AChE.

In conclusion, an Amaryllidaceae alkaloid with a rare imidazo[1,2-*f*]phenanthridine framework, zephycandidine A (**1**), was isolated from *Z. candida*. Zephycandidine A (**1**), [1,3]dioxolo[4,5-*j*]imidazo[1,2-*f*]phenanthridine, represents the first example of a naturally occurring imidazo[1,2-*f*]phenanthridine type alkaloid. Zephycandidine A (**1**) exhibited cytotoxicity against five cancer cell lines with IC_50_ values ranging from 1.98 to 7.03 *μ*M with selectivity indices as high as 10 when compared to the normal Beas-2B cells. More importantly, zephycandidine A (**1**) induced apoptosis in leukemia cells by the activation of caspase-3, upregulation of Bax, downregulation of Bcl-2 and degradation of PARP expression. Zephycandidine A (**1**) showed AChE inhibitory activity with IC_50_ value of 127.99 μM, and the docking studies of **1** with AChE revealed that interactions with W286 and Y337 are necessary.

## Methods

### General experimental procedures

The UV and FT-IR spectra were determined using Varian Cary 50 and Bruker Vertex 70 instruments, respectively. NMR spectra were recorded with a Bruker AM-400 spectrometer for ^1^H NMR, ^13^C NMR, COSY, HSQC, HMBC, and NOESY experiments. Chemical shift values are given in *δ* (ppm) based on the peak signals of the solvent methanol-*d*_4_ (*δ*_H_ 3.31 and *δ*_C_ 49.15) as references, and coupling constants are reported in Hz. High-resolution electrospray ionization mass spectra (HRESIMS) were carried out in the positive-ion mode on a Thermo Fisher LC-LTQ-Orbitrap XL spectrometer. The OD values were measured on a Bio-Rad 680 microtiter plate reader. HPLC was performed on an Agilent 2100 quaternary system with a UV detector using a reversed-phased C_18_ column (5 *μ*m, 10 × 250 mm, Weltch Ultimate XB-C18). Column chromatography was performed using Sephadex LH-20 (GE Healthcare Bio-Sciences AB, Sweden), silica gel (Qingdao Marine Chemical Inc., China), and ODS (50 *μ*m, YMC Co. Ltd., Japan). Thin-layer chromatography (TLC) was performed with silica gel 60 F254 (Yantai Chemical Industry Research Institute) and RP-C_18_ F254 plates (Merck, Germany).

### Plant material

The whole plants of *Zephyranthes candida* were collected at Shiyan, Hubei Province, People’s Republic of China, in October, 2011. The plant material was identified by Professor Changgong Zhang of the School of Pharmacy, Tongji Medical College, Huazhong University of Science and Technology. A voucher specimen (No. 20111001) has been deposited at the Hubei Key Laboratory of Natural Medicinal Chemistry and Resource Evaluation, School of Pharmacy, Tongji Medical College, Huazhong University of Science and Technology.

### Extraction and isolation

The whole dried plants of *Z. candida* (10 kg) were extracted four times with 25 L each of 95% aqueous EtOH with 2% HCl at room temperature. The filtrates were combined and concentrated under vacuum to afford 627 g of crude extract, which was then partitioned between CHCl_3_ and adjusted the pH = 2 with dilute HCl, then extracted the aqueous phase with CHCl_3_, followed by re-extracting the aqueous phase three additional times with CHCl_3_ (3 L). After the aqueous phase was adjusted to pH 10 with an ammonia solution, it was partitioned between CHCl_3_ (4 × 1.5 L) for the second time. On evaporation, the CHCl_3_ phase at pH = 10 (7.6 g) was subjected to silica gel CC (CHCl_3_–MeOH, 1:0, 50:1, 30:1, 20:1, 10:1) to afford 5 fractions (Fr. 1–5). Fr.2 was fractionated on MPLC with RP-18 CC (MeOH–H_2_O, 60:40, 90:10) to give four subfractions (Fr. 2A–Fr.2D). Fr.2C was subjected to separation over silica gel using a gradient system of petroleum ether–acetone (10:1–2:1) to provide Fr.2CA. Compound **1** (3.5 mg, *t*_R_ 23.8 min) were purified from the Fr.2CA, using semi-preparative RP HPLC (MeOH–H_2_O–Et_2_NH, 80:20:0.2).

#### Zephycandidine A (**1**)

colorless oil; UV (MeOH) *λ*_max_ (log *ε*) 207 (4.63), 220 (4.48), 256 (3.63), 297 (4.18) nm; IR (KBr) *ν*_max_ 2920, 2851, 1622, 1486, 1383, 1258, 1208, 1037, 886, 829, 750, 721 cm^−1^; ^1^H NMR data, see Table 1; ^13^C NMR data, see Table 1; HRESIMS *m/z* 263.0789 [M + H]^+^ (calcd for C_16_H_11_N_2_O_2_, 263.0821).

### Cytotoxicity assay

Cytotoxity of the compound against five human cancer cell lines, human myeloid leukemia HL-60, lung cancer A549, breast cancer MCF-7, colon cancer SW480, and hepatocellular carcinoma SMMC-7721, together with one non-cancerous cell line human bronchial epithelial cells Beas-2B, was evaluated using the MTT method^10^. Briefly, the cells were grown in RPMI 1640 (Hyclone) medium supplemented with 10% heat-inactivated newborn calf serum, penicillin (50 units/mL), and streptomycin (50 g/mL) in a humidified incubator under 5% CO_2_ at 37 °C. Cell suspensions (200 μL, containing 5000–10000 cells per well) were placed into 96 well microplates and allowed to adhere for 24 h before drug addition, while suspended cells were seeded just before drug addition. A 100 μL aliquot of the test compounds at concentrations ranging from 2.5 to 40 μM was added to each well. The medium was replaced with one containing the test compounds, and the cells were further cultured at 37 °C. After incubation for 48 h, 20 μL of MTT (5 mg/mL) solution was added to each well and the cells were incubated under the same conditions for 4 h until a purple precipitate was visible. DMSO (200 mL) was added and the optical density was measured at 570 nm in a microplate reader (Bio-Tek Synergy HT). DDP (*cis*-platin) and DMSO were used as the positive and negative controls, respectively.

### Cell morphological assessment

The cell morphological assessment was performed after HL-60 cells were treated with 0, 3 and 6 μM of zephycandidine A (**1**) for 48 hours. Cell morphological changes were observed with inverted light microscopy (NIKON, Tokyo, Japan).

### Flow cytometry analysis of cell apoptosis

For identification of the apoptotic induction effect of zephycandidine A (**1**), a FITC-labeled Annexin V/PI apoptosis detection Kit (Keygen, Nanjing, China) was used according to the manufacturer’s instructions. Briefly, HL-60 cells were exposure to vehicle control (DMSO, < 0.1%), zephycandidine A (**1**) (3 and 6 μM) for 48 h, VP16 was used as a positive control^20^. After that cells were harvested and washed with PBS and resuspended in binding buffer, and then, AnnexinV-FITC and PI were added. After staining 15 minutes, the cells were immediately analyzed using flow cytometry (Becton Dickinson, USA).

### Western blot analysis

Western blot analysis was conducted to analysis the protein expression. Briefly, cells were incubated with vehicle control and zephycandidine A (**1**) for 48 hours, and then cells were lysed in a radio immune-precipitation assay buffer. Protein concentrations were determined and equalized before loading. Samples containing equal amounts of protein were denatured and subjected to electrophoresis in 10% SDS-PAGE gels followed by transfer to PVDF membrane and probed with specific antibodies, including PARP, Bcl-2, Bax, Cleaved Caspase 3 and *β*-Actin (Cell Signaling Technology, Inc.). Blots bands were visualized using the horseradish peroxidase conjugated secondary antibodies and chemiluminescent substrate.

### Acetylcholinesterase inhibitory activity assay

AChE activity was measured using a 96-well microplate reader based on Ellman’s method^14^ with slight modification^15^. The enzyme hydrolyzes the substrate acetylthiocholine resulting in the product thiocholine which reacts with Ellman’s reagent (DTNB) to produce a yellow compound 5-thio-2-nitrobenzoate which can be detected at 405 nm. All solutions were brought to room temperature prior to use. In a typical assay, 25 *μ*L of 15 m*M* ATCI (dissolved in water), 125 *μ*L of 3 mM DTNB (50 mM Tris–HCl, pH 7.6, containing 0.1 *M* NaCl and 0.02 *M* MgCl_2_•6 H_2_O), 50 *μ*L of buffer (50 mM Tris–HCl, pH 7.6, containing 0.1% BSA), 25*μ*L of sample were added in the 96-well plates and the absorbance was measured at 405 nm every minute for six times. Then, 25 *μ*L of 0.22 U/ml of enzyme was added. Read the absorbance again every minute for fifteen times. The rates of reaction were calculated by Microplate Manager software version 4.0. Any increase in absorbance due to the spontaneous hydrolysis of substrate was corrected by subtracting the rate of the reaction before adding the enzyme from the rate after adding the enzyme. Percentage of inhibition was calculated by comparing the rates for the sample to the blank (10% MeOH in buffer). The inhibition rate of zephycandidine A (**1**) against AChE was initially evaluated at the concentration of 200 *μ*M. Based on the inhibition rate at 200 *μ*M, the test concentrations of the active compounds were further optimized for IC_50_ calculation.

### NMR data calculations

The 3D structure of the compound **1a** and **1b** were optimized in methanol by using Gaussian09 at the B3LYP/6-31G* level. Both optimized structures (Tables S1 and S2, Supplementary Information) were then further used as the input structures for NMR calculation. The NMR calculations were performed by using Gaussian09 at the B3LYP/6-31G* level. Finally, the relative errors between the computed (Tables S3–S6, Supplementary Information) and measured NMR were calculated^21^,^22^ (Figures S1–S6, Supplementary Information).

### Docking Studies

The docking studies were performed by using Autodock 4.2^23^ in combination with MM/GBSA method implemented in AMBER 11^24^. The basic idea of this combination method is to replace the scoring function used in Autodock by the more expensive but more accurate MM/GBSA energy. Therefore, only 1 generation was evolved within the Genetic Algorithm, and the wide spread docking poses were produced as many as 10,000 by the Autodock software. All conformations were subjected to MM/GBSA calculations, and then were ranked according the much more reliable MM/GBSA results.

To evaluate the accuracy of our docking method, five crystal structures^23^,^25^ (PDB IDs: 4EY5, 4EY6, 4EY7, 4M0E, 4M0F) with ligands in complex with human AChE were used for re-docking tests. The initial coordinates of ligands were not taken from the crystal structures, but generated by BALLON software^26^ in order to avoid the bias from Autodock software to the initial structure. The top 1 ranked binding structures obtained in our docking method are all having RMSD less than 2 Å as compared to the binding poses in crystal structures, indicating that our docking method is very reliable in reproducing the correct binding poses for AChE inhibitors.

## Additional Information

**How to cite this article**: Zhan, G. *et al.* Zephycandidine A, the First Naturally Occurring Imidazo[1,2-*f* ]phenanthridine Alkaloid from *Zephyranthes candida*, Exhibits Significant Anti-tumor and Anti-acetylcholinesterase Activities. *Sci. Rep.* **6**, 33990; doi: 10.1038/srep33990 (2016).

## Supplementary Material

Supplementary Information

## Figures and Tables

**Figure 1:**
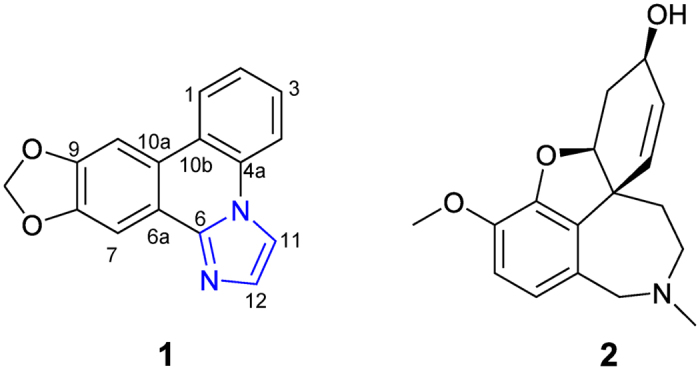
Chemical structures of zephycandidine A (**1**) and galanthamine (**2**).

**Table 1:** ^1^H (400 MHz) and ^13^C NMR (100 MHz) data for zephycandidine A (**1**) (Methanol-*d*_4_) and DFT calculation of ^13^C NMR for **1a** and **1b**.

position	1		1a		1b	
	*δ*_H_ (mult, *J*)	*δ*_C_	*δ*_C_(cal.)	*δ*_C_(cor.)	*δ*_C_(cal.)	*δ*_C_(cor.)
1	8.47, dd, (8.2, 1.2)	125.4	128.6	125.4	128.6	126.2
2	7.58, ddd, (8.2, 7.4, 1.0)	126.9	129.1	125.9	129.8	127.7
3	7.67, ddd, (8.4, 7.4, 1.2)	129.8	131.8	128.9	131.9	130.3
4	8.14, dd, (8.4, 1.0)	117.5	120.9	116.9	120.3	115.8
4a		132.3	135.4	132.9	134.4	133.5
6		143.8	145.5	144.0	132.8	131.5
6a		119.8	125.0	121.4	125.2	121.9
7	7.88, s	103.2	107.8	102.5	107.3	99.5
8		150.7	151.8	150.9	151.9	155.4
9		151.6	152.3	151.4	151.3	154.6
10	7.99, s	103.1	106.8	101.4	107.4	99.6
10a		125.5	128.1	124.8	126.1	123.1
10b		123.1	127.3	123.9	127.9	125.3
11	7.52, d, (1.4)	113.8	116.8	112.4	131.5	129.8
12	8.32, d, (1.4)	131.5	134.7	132.1	125.3	122.1
OCH_2_O	6.16, s	103.7	111.8	106.9	111.8	105.2

**Table 2:** Cytotoxicity of zephycandidine A (1) (IC_50_ in *μ*M) against five cancer cell lines and a non-cancerous human Beas-2B cell line^a^.

Compound	HL-60	A549	MCF-7	SW480	SMMC-7721	BEAS-2B	Highest index of selectivity
**1**	1.98	6.49	3.44	6.27	7.03	20.08	10
***cis*****-platin**	3.12	8.95	18.48	11.37	9.82	13.30	4.3

^a^The “Highest index of selectivity” is the ratio of the IC_50_ value for the Beas-2B cell line over the lowest cancer cell IC_50_ value. *cis*-Platin was used as a positive control.
