# Supplementary material for: Zephycandidine A, the First Naturally Occurring Imidazo[1,2-f]phenanthridine Alkaloid from Zephyranthes candida, Exhibits Significant Anti-tumor and Anti-acetylcholinesterase Activities
Source: Sci Rep. 2016 Sep 23;6:33990. doi: 10.1038/srep33990 (PMC5034291; doi:10.1038/srep33990)
Supplement: Supplementary Information [file srep33990-s1.pdf]

# Supplementary Information

# Zephycandidine A, the First Naturally Ocurring Imidazo[1,2-*f*]phenanthridine Alkaloid from *Zephyranthes candida*, Exhibits Significant Anti-tumor and Anti-acetylcholinesterase Activities

**Guanqun Zhan[1,#], Xiaolan Qu[2,#], Junjun Liu[1], Qingyi Tong[1], Junfei Zhou[1] , Bin Sun[1] & Guangmin Yao[1,*]**

[1]Hubei Key Laboratory of Natural Medicinal Chemistry and Resource Evaluation, School of Pharmacy, Tongji Medical College, Huazhong University of Science and Technology, Wuhan 430030, People's Republic of China.

[2]School of Pharmaceutical Sciences, Taishan Medical University, Tai-An 271016, People's Republic of China.

*Correspondence and requests for materials should be addressed to G.Y. (gyap@mail.hust.edu.cn)

[#]These authors contributed equally to this work.

# Contents

**Table S1.** The optimized structure in methanol of compound **1a** at B3LYP/6-31G* ........ 3
**Table S2.** The optimized structure in methanol of compound **1b** at B3LYP/6-31G* ........ 4
**Table S3.** DFT Calculation Result for $\delta_H$ shifts of **1a** ........ 5
**Figure S1.** The $^1$H NMR correlation of experimental data and calculated dada about **1a** ........ 5
**Table S4.** DFT Calculation Result for $\delta_H$ shifts of **1b** ........ 6
**Figure S2.** The $^1$H NMR correlation of experimental data and calculated dada about **1b** ........ 6
**Figure S3.** Comparing DFT calculated vs. experimental $^1$H $\delta$ values for two possible structures........ 7
**Table S5.** DFT Calculation Result for C shifts of **1a** ........ 7
**Figure S4.** The $^{13}$C NMR correlation of experimental data and calculated dada about **1a** ........ 8
**Table S6.** DFT Calculation Result for C shifts of **1b** ........ 8
**Figure S5.** The $^{13}$C NMR correlation of experimental data and calculated dada about **1b** ........ 9
**Figure S6.** Comparing DFT calculated vs. experimental $^{13}$C $\delta$ values for two possible structures. ........ 9
**Figure S7.** (+)-HRESI-MS spectrum of **1** ........ 10
**Figure S8.** UV spectrum of **1** (MeOH) ........ 10
**Figure S9.** IR spectrum of **1** (KBr disc) ........ 11
**Figure S10.** $^1$H NMR spectrum of **1** (CD3OD, 400 MHz) ........ 11
**Figure S11.** $^{13}$C NMR spectrumof **1** (CD3OD, 100 MHz) ........ 12
**Figure S12.** DEPT spectrum of **1** ($CD_3OD$, 100 MHz) ........ 12
**Figure S13.** HSQC spectrum of **1** ($CD_3OD$) ........ 13
**Figure S14.** HMBC spectrum of **1** ($CD_3OD$) ........ 13
**Figure S15.** $^1$H–$^1$H COSY spectrum of **1** ($CD_3OD$) ........ 14
**Figure S16.** NOESY spectrum of **1** ($CD_3OD$) ........ 14
**Figure S17.** Original blots of Figure 5C. ........ 15

**Table S1.** The optimized structure in methanol of compound **1a** at B3LYP/6-31G*

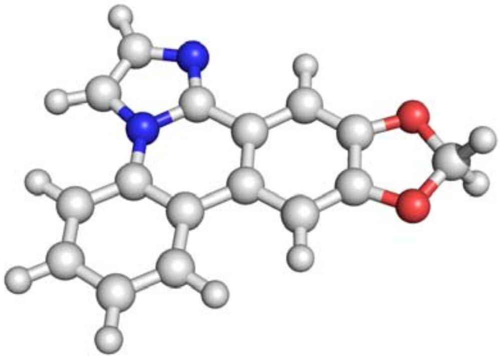

| | x | y | z |
|---|---|---|---|
| C | 1.381400 | -1.441116 | -0.000004 |
| C | 2.619630 | -0.850787 | -0.000013 |
| C | 2.788710 | 0.540815 | -0.000030 |
| C | 1.717087 | 1.394627 | -0.000038 |
| C | 0.424531 | 0.810093 | -0.000026 |
| C | 0.237742 | -0.595503 | -0.000012 |
| C | -0.733288 | 1.667865 | -0.000016 |
| N | -2.005026 | 1.119254 | 0.000014 |
| C | -2.244044 | -0.264140 | 0.000012 |
| C | -1.125989 | -1.136943 | -0.000005 |
| C | -3.555565 | -0.761770 | 0.000021 |
| C | -3.780210 | -2.130653 | 0.000012 |
| C | -2.693679 | -3.015487 | -0.000011 |
| C | -1.397716 | -2.522341 | -0.000020 |
| O | 3.844678 | -1.461911 | -0.000031 |
| C | 4.824684 | -0.410928 | 0.000122 |
| O | 4.122504 | 0.843427 | -0.000058 |
| N | -0.774249 | 2.995295 | -0.000024 |
| C | -2.110542 | 3.311144 | 0.000029 |
| C | -2.893456 | 2.183970 | 0.000026 |
| H | 1.299687 | -2.520322 | 0.000012 |
| H | 1.829065 | 2.471930 | -0.000050 |
| H | -4.396348 | -0.077667 | 0.000035 |
| H | -4.798007 | -2.508633 | 0.000021 |
| H | -2.861926 | -4.088156 | -0.000023 |
| H | -0.575490 | -3.228570 | -0.000043 |
| H | 5.437445 | -0.484498 | 0.903506 |
| H | 5.437788 | -0.484561 | -0.903019 |
| H | -2.447592 | 4.339642 | 0.000047 |
| H | -3.962925 | 2.053791 | 0.000035 |

HF=-875.689933532

**Table S2.** The optimized structure in methanol of compound **1b** at B3LYP/6-31G*

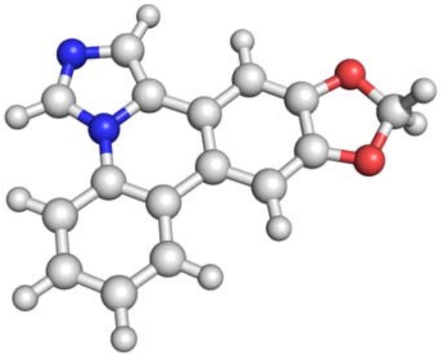

| | x | y | z |
|---|---|---|---|
| C | 2.633564 | -0.841085 | -0.000068 |
| C | 2.799041 | 0.548547 | 0.000047 |
| C | 1.724615 | 1.401139 | 0.000098 |
| C | 0.429420 | 0.821441 | 0.000037 |
| C | 0.250696 | -0.585558 | -0.000052 |
| C | 1.394329 | -1.429766 | -0.000117 |
| C | -0.734902 | 1.673823 | 0.000035 |
| N | -2.011865 | 1.111321 | -0.000012 |
| C | -2.238246 | -0.278476 | 0.000010 |
| C | -1.112678 | -1.138773 | -0.000038 |
| C | -3.544372 | -0.786679 | 0.000091 |
| C | -3.757623 | -2.158482 | 0.000094 |
| C | -2.663750 | -3.031898 | 0.000028 |
| C | -1.371684 | -2.525694 | -0.000029 |
| C | -0.944705 | 3.040808 | -0.000010 |
| N | -2.287336 | 3.324119 | -0.000086 |
| C | -2.902641 | 2.157282 | -0.000086 |
| O | 3.860816 | -1.450528 | -0.000129 |
| C | 4.837294 | -0.396853 | 0.000081 |
| O | 4.131190 | 0.856334 | 0.000071 |
| H | 1.858746 | 2.476581 | 0.000181 |
| H | 1.312791 | -2.508951 | -0.000205 |
| H | -4.392624 | -0.111870 | 0.000159 |
| H | -4.772154 | -2.544961 | 0.000156 |
| H | -2.820899 | -4.106251 | 0.000033 |
| H | -0.543892 | -3.225179 | -0.000059 |
| H | -0.202511 | 3.827434 | -0.000003 |
| H | -3.972475 | 2.015283 | -0.000150 |
| H | 5.450738 | -0.467572 | -0.903097 |
| H | 5.450491 | -0.467698 | 0.903421 |

HF=-875.684718638

**Table S3.** DFT Calculation Result for $\delta_H$ shifts of **1a**

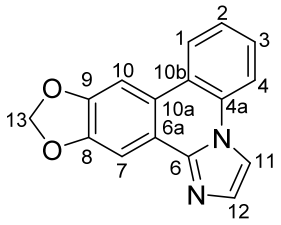

**1a**

| Carbon Number | Chemical Shift | Calculated Shift | Corrected | difference | \|difference\| |
|---|---|---|---|---|---|
| 1 | 8.47 | 8.63 | 8.61 | 0.14 | 0.14 |
| 2 | 7.58 | 7.97 | 7.73 | 0.15 | 0.15 |
| 3 | 7.67 | 8.06 | 7.85 | 0.18 | 0.18 |
| 4 | 8.14 | 8.31 | 8.18 | 0.04 | 0.04 |
| 7 | 7.88 | 8.15 | 7.97 | 0.09 | 0.09 |
| 10 | 7.99 | 7.88 | 7.61 | -0.38 | 0.38 |
| 11 | 7.52 | 7.79 | 7.49 | -0.03 | 0.03 |
| 12 | 8.32 | 8.31 | 8.18 | -0.14 | 0.14 |
| $OCH_2O$ | 6.16 | 6.75 | 6.11 | -0.05 | 0.05 |
| | | | | average | 0.13 |
| | | | | max | 0.38 |

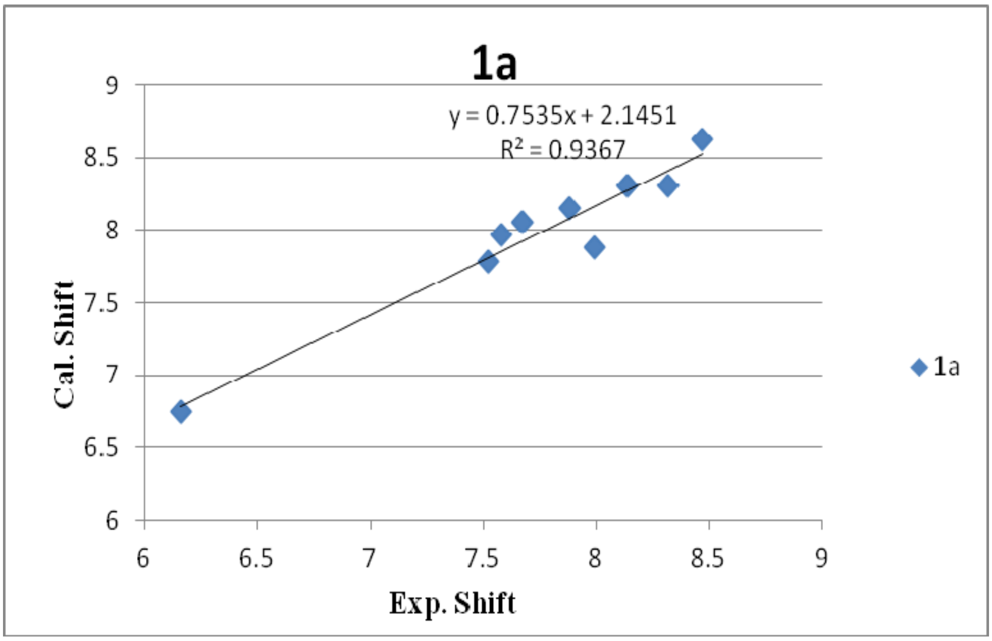


**Figure S1.** The $^{1}$H NMR correlation of experimental data and calculated dada about **1a**

**Table S4.** DFT Calculation Result for $\delta_H$ shifts of **1b**

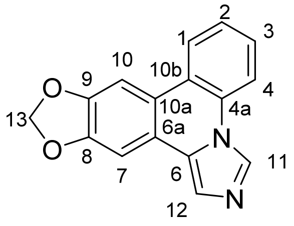


| Carbon Number | Chemical Shift | Calculated Shift | Corrected | difference | \|difference\| |
|---|---|---|---|---|---|
| 1 | 8.47 | 8.47 | 8.43 | -0.04 | 0.04 |
| 2 | 7.58 | 7.94 | 7.75 | 0.17 | 0.17 |
| 3 | 7.67 | 7.94 | 7.75 | 0.08 | 0.08 |
| 4 | 8.14 | 8.37 | 8.30 | 0.16 | 0.16 |
| 7 | 7.88 | 7.58 | 7.29 | -0.59 | 0.59 |
| 10 | 7.99 | 7.76 | 7.52 | -0.47 | 0.47 |
| 11 | 7.52 | 8.75 | 8.79 | 1.27 | 1.27 |
| 12 | 8.32 | 7.94 | 7.75 | -0.57 | 0.57 |
| $OCH_2O$ | 6.16 | 6.71 | 6.17 | 0.01 | 0.01 |
| | | | | average | 0.37 |
| | | | | max | 1.27 |

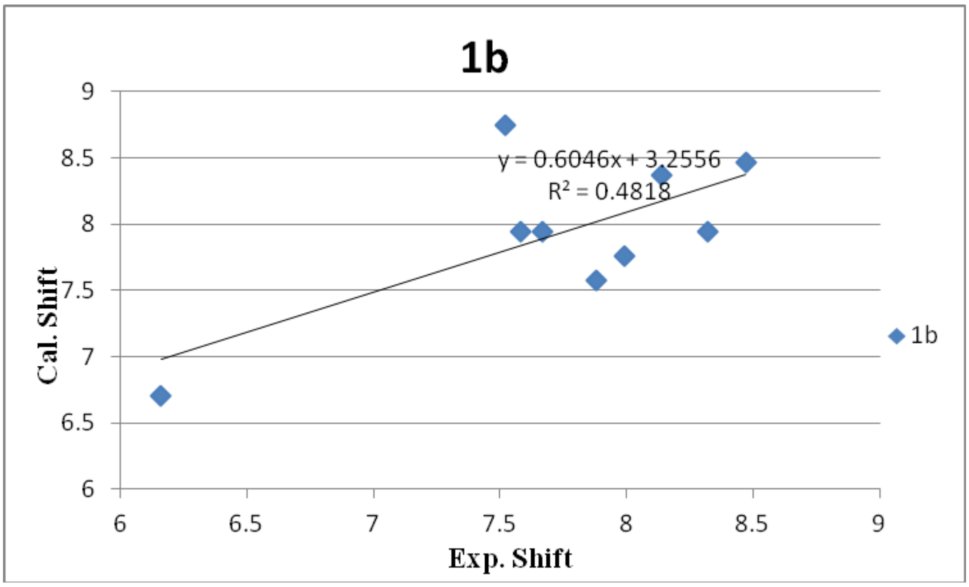


**Figure S2.** The $^1$H NMR correlation of experimental data and calculated dada about **1b**

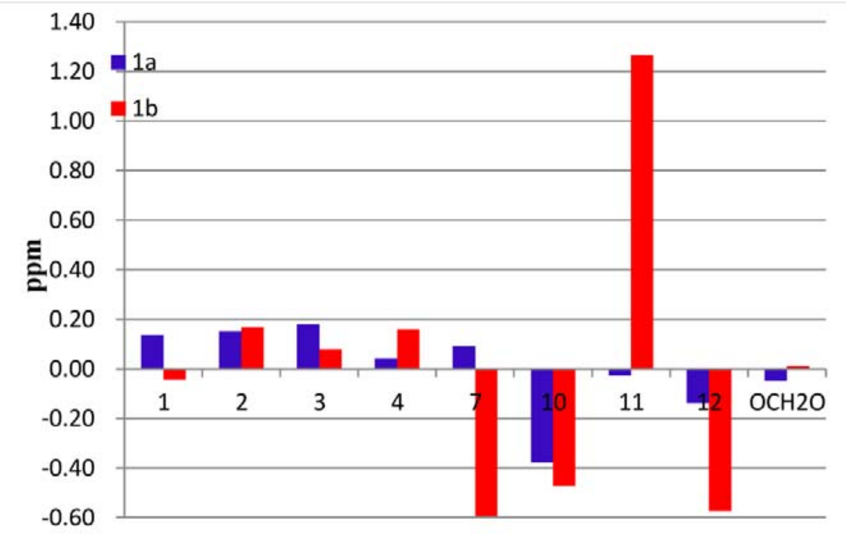


**Figure S3.** Comparing DFT calculated vs. experimental [1]H $\delta$ values for two possible structures.

**Table S5.** DFT Calculation Result for C shifts of **1a**

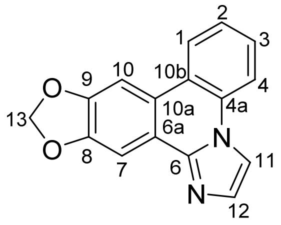


**1a**

| Carbon Number | Chemical Shift | Calculated Shift | Corrected | difference | \|difference\| |
|---|---|---|---|---|---|
| 1 | 125.4 | 128.6 | 125.4 | 0.0 | 0.0 |
| 2 | 126.9 | 129.1 | 125.9 | -1.0 | 1.0 |
| 3 | 129.8 | 131.8 | 128.9 | -0.9 | 0.9 |
| 4 | 117.5 | 120.9 | 116.9 | -0.6 | 0.6 |
| 4a | 132.3 | 135.4 | 132.9 | 0.6 | 0.6 |
| 6 | 143.8 | 145.5 | 144.0 | 0.2 | 0.2 |
| 6a | 119.8 | 125.0 | 121.4 | 1.6 | 1.6 |
| 7 | 103.2 | 107.8 | 102.5 | -0.7 | 0.7 |
| 8 | 150.7 | 151.8 | 150.9 | 0.2 | 0.2 |
| 9 | 151.6 | 152.3 | 151.4 | -0.2 | 0.2 |
| 10 | 103.1 | 106.8 | 101.4 | -1.7 | 1.7 |
| 10a | 125.5 | 128.1 | 124.8 | -0.7 | 0.7 |
| 10b | 123.1 | 127.3 | 123.9 | 0.8 | 0.8 |
| 11 | 113.8 | 116.8 | 112.4 | -1.4 | 1.4 |
| 12 | 131.5 | 134.7 | 132.1 | 0.6 | 0.6 |
| 13 | 103.7 | 111.8 | 106.9 | 3.2 | 3.2 |
| | | | | average | 0.9 |
| | | | | max | 3.2 |

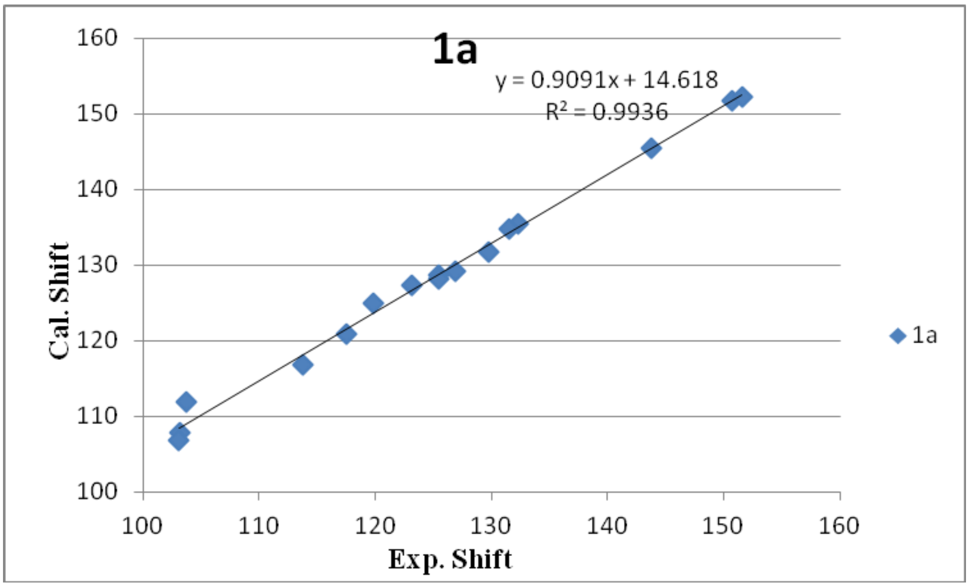


**Figure S4.** The $^{13}$C NMR correlation of experimental data and calculated dada about **1a**

**Table S6.** DFT Calculation Result for C shifts of **1b**

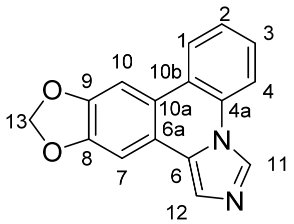


**1b**

| Carbon Number | Chemical Shift | Calculated Shift | Corrected | difference | \|difference\| |
|---|---|---|---|---|---|
| 1 | 125.4 | 128.6 | 126.2 | 0.8 | 0.8 |
| 2 | 126.9 | 129.8 | 127.7 | 0.8 | 0.8 |
| 3 | 129.8 | 131.9 | 130.3 | 0.5 | 0.5 |
| 4 | 117.5 | 120.3 | 115.8 | -1.7 | 1.7 |
| 4a | 132.3 | 134.4 | 133.5 | 1.2 | 1.2 |
| 6 | 143.8 | 132.8 | 131.5 | -12.3 | 12.3 |
| 6a | 119.8 | 125.2 | 121.9 | 2.1 | 2.1 |
| 7 | 103.2 | 107.3 | 99.5 | -3.7 | 3.7 |
| 8 | 150.7 | 151.9 | 155.4 | 4.7 | 4.7 |
| 9 | 151.6 | 151.3 | 154.6 | 3.0 | 3.0 |
| 10 | 103.1 | 107.4 | 99.6 | -3.5 | 3.5 |
| 10a | 125.5 | 126.1 | 123.1 | -2.4 | 2.4 |
| 10b | 123.1 | 127.9 | 125.3 | 2.2 | 2.2 |
| 11 | 113.8 | 131.5 | 129.8 | 16.0 | 16.0 |
| 12 | 131.5 | 125.3 | 122.1 | -9.4 | 9.4 |
| 13 | 103.7 | 111.8 | 105.2 | 1.5 | 1.5 |
| | | | | average | 4.1 |
| | | | | max | 16.0 |

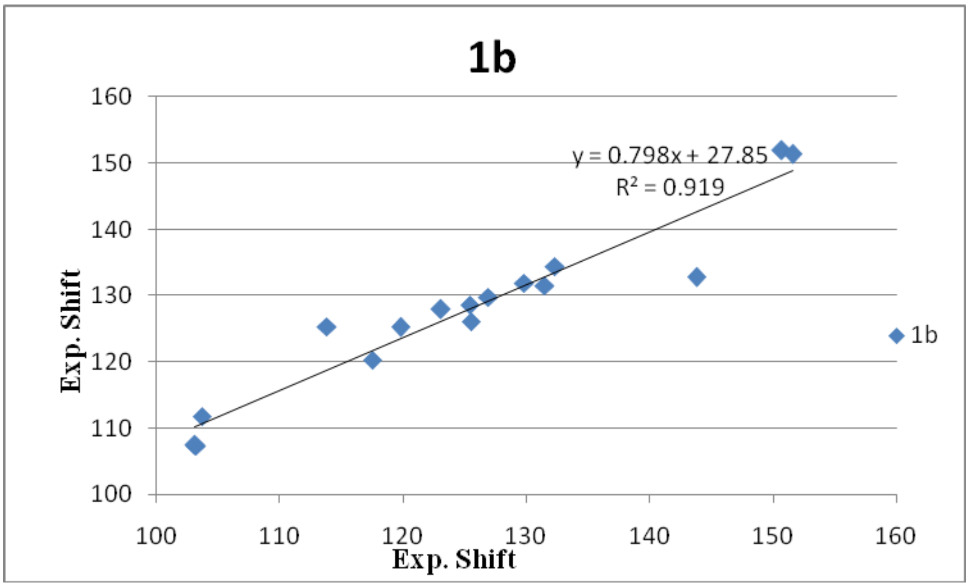


**Figure S5.** The $^{13}$C NMR correlation of experimental data and calculated dada about **1b**

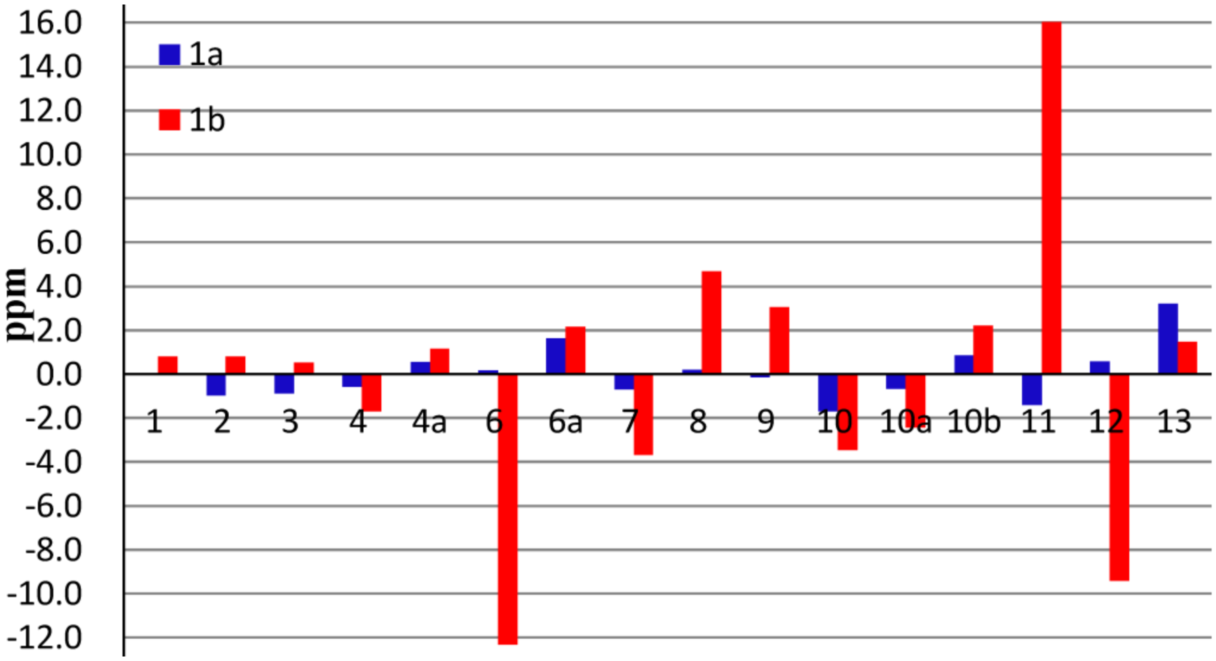


**Figure S6.** Comparing DFT calculated vs. experimental $^{13}$C $\delta$ values for two possible structures.

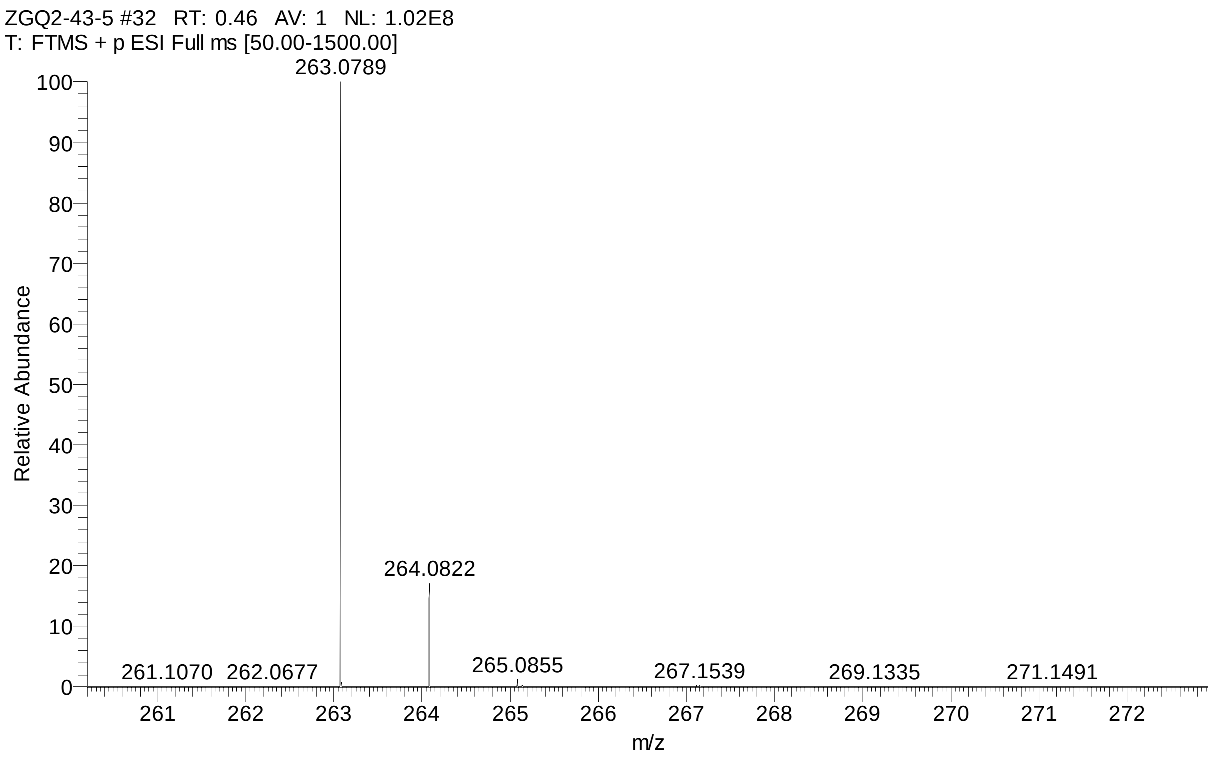


**Figure S7.** (+)-HRESI-MS spectrum of **1**

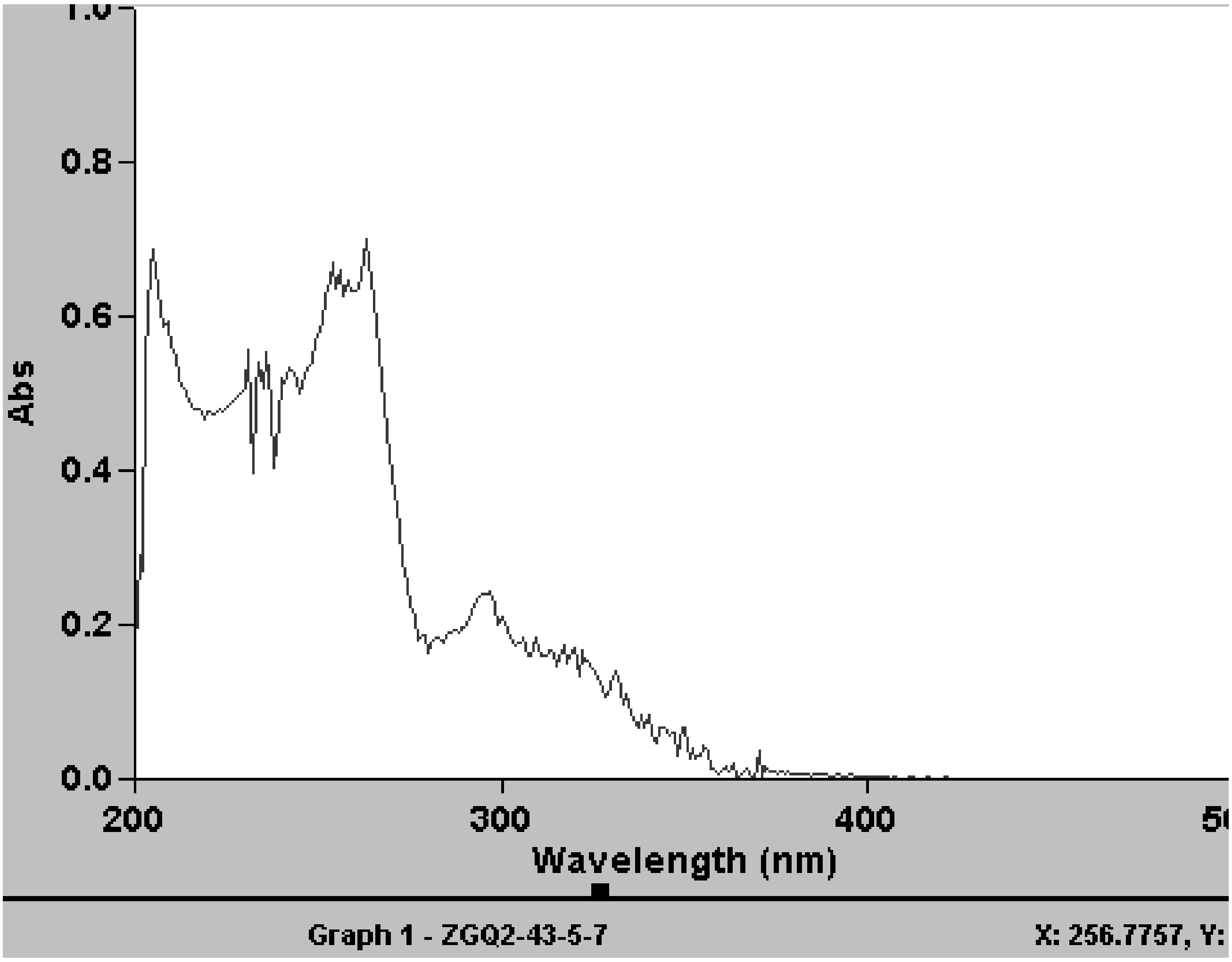


**Figure S8.** UV spectrum of **1** (MeOH)

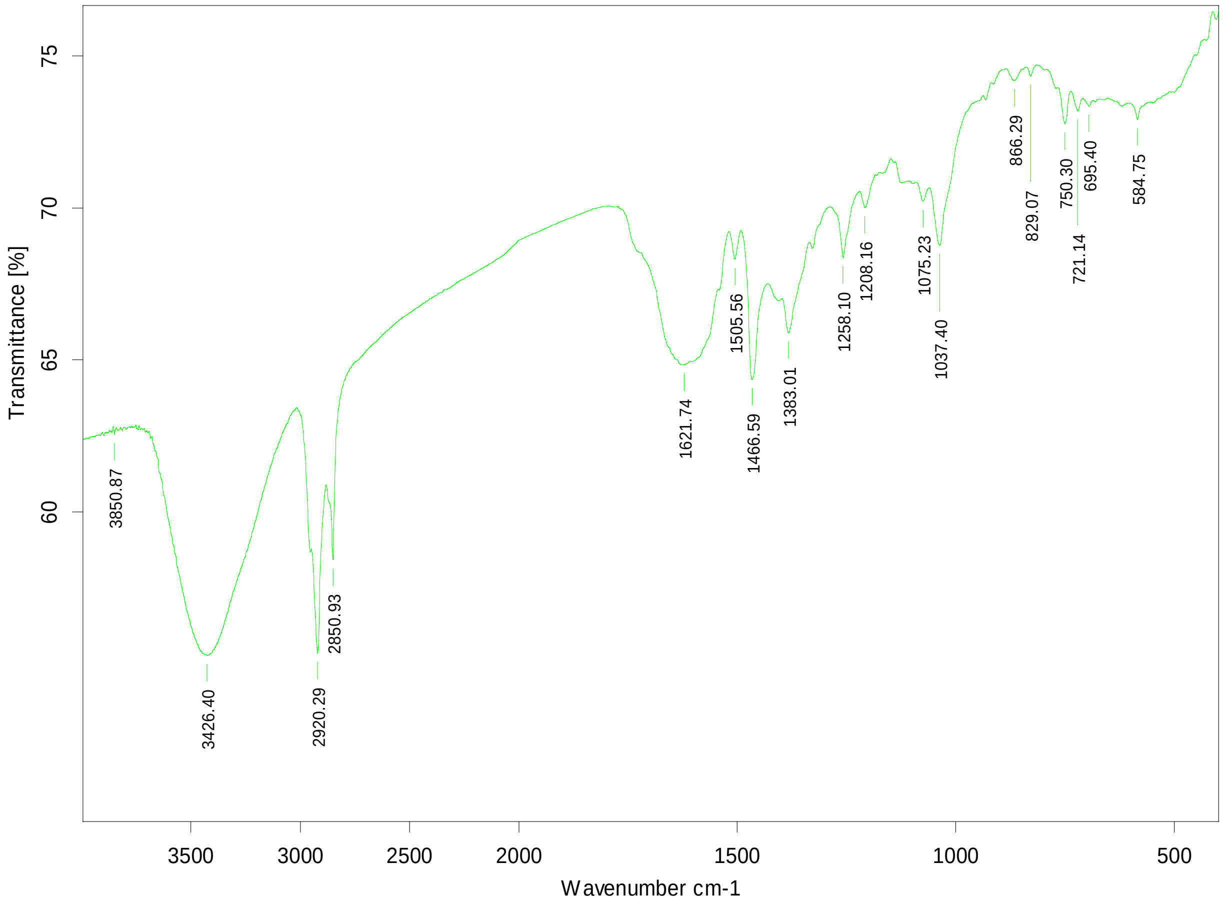


**Figure S9.** IR spectrum of **1** (KBr disc)

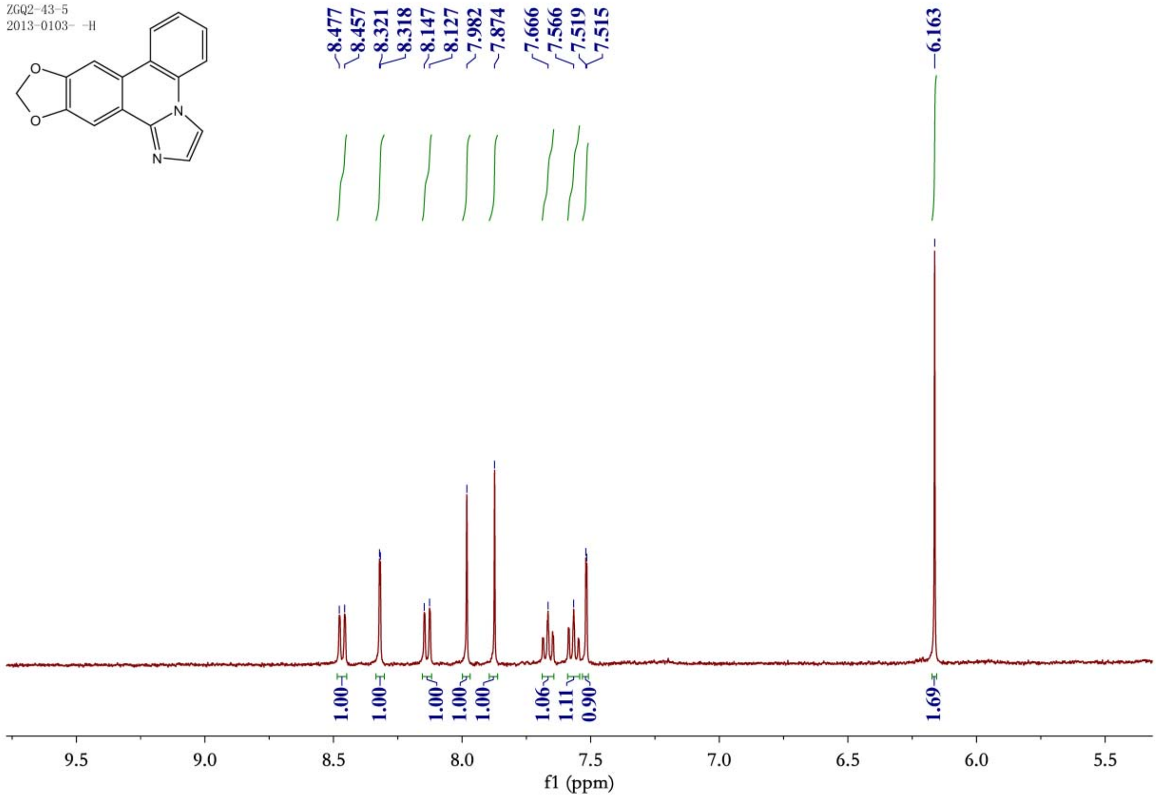


**Figure S10.** $^{1}$H NMR spectrum of **1** (CD3OD, 400 MHz)

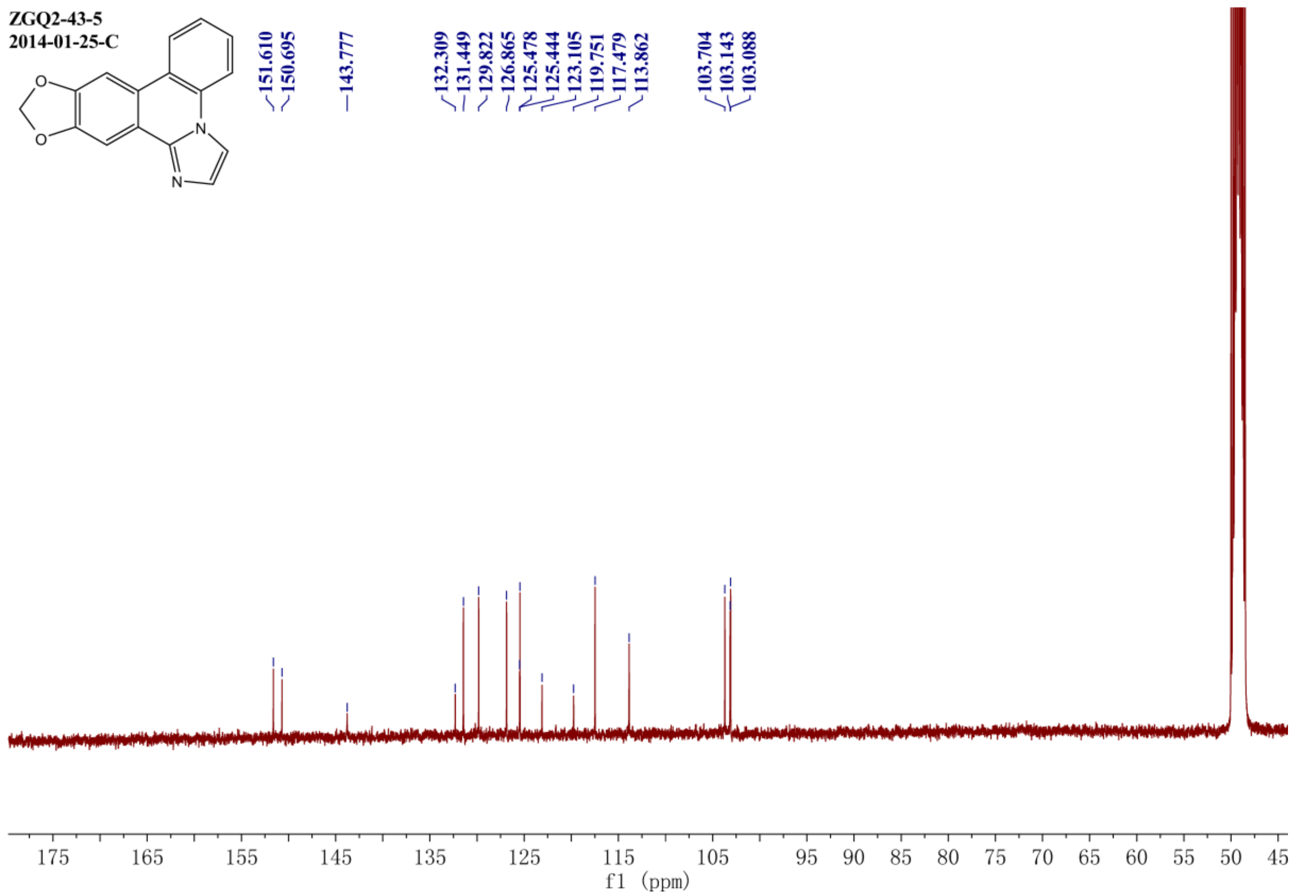


**Figure S11.** $^{13}$C NMR spectrumof **1** (CD3OD, 100 MHz)

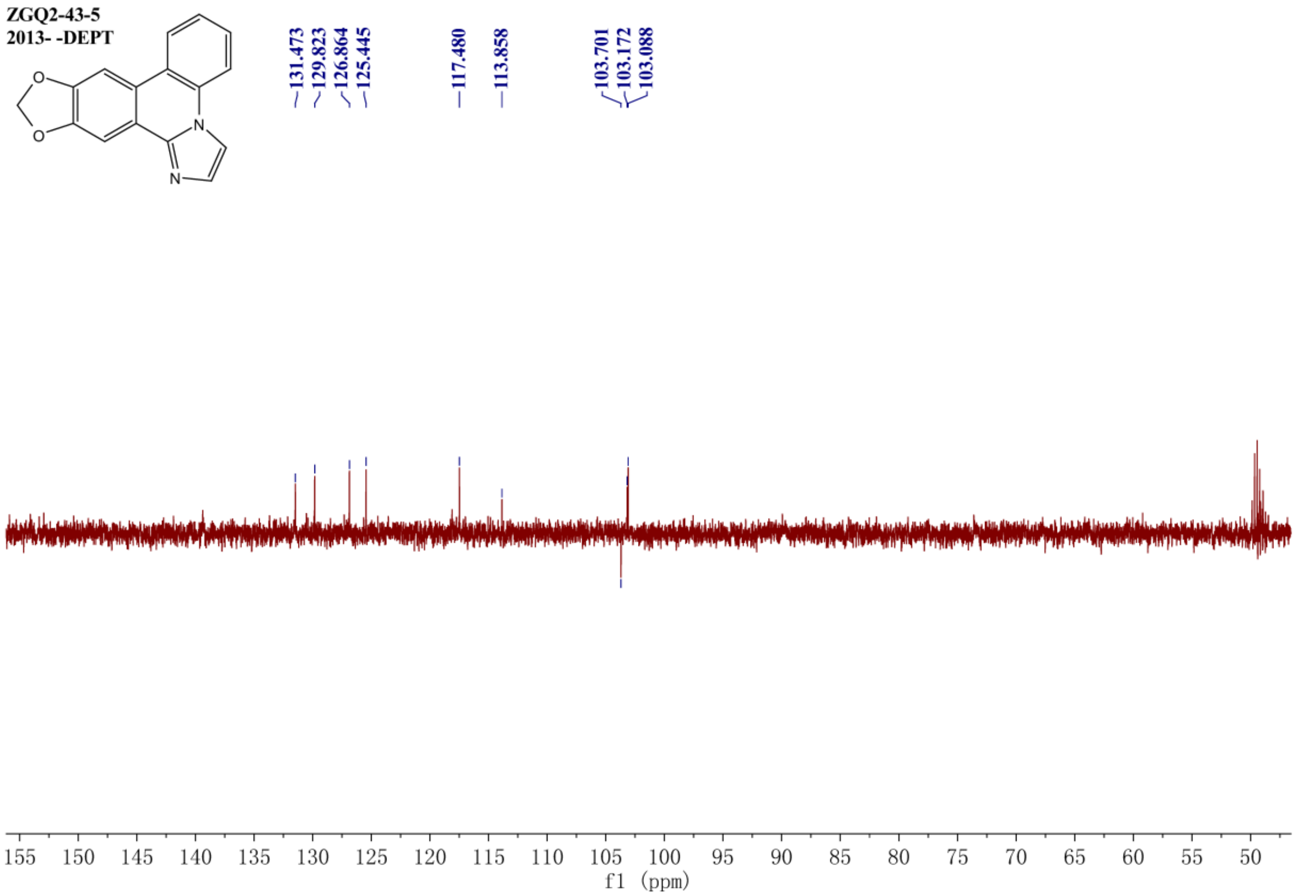


**Figure S12.** DEPT spectrum of **1** ($CD_3OD$, 100 MHz)

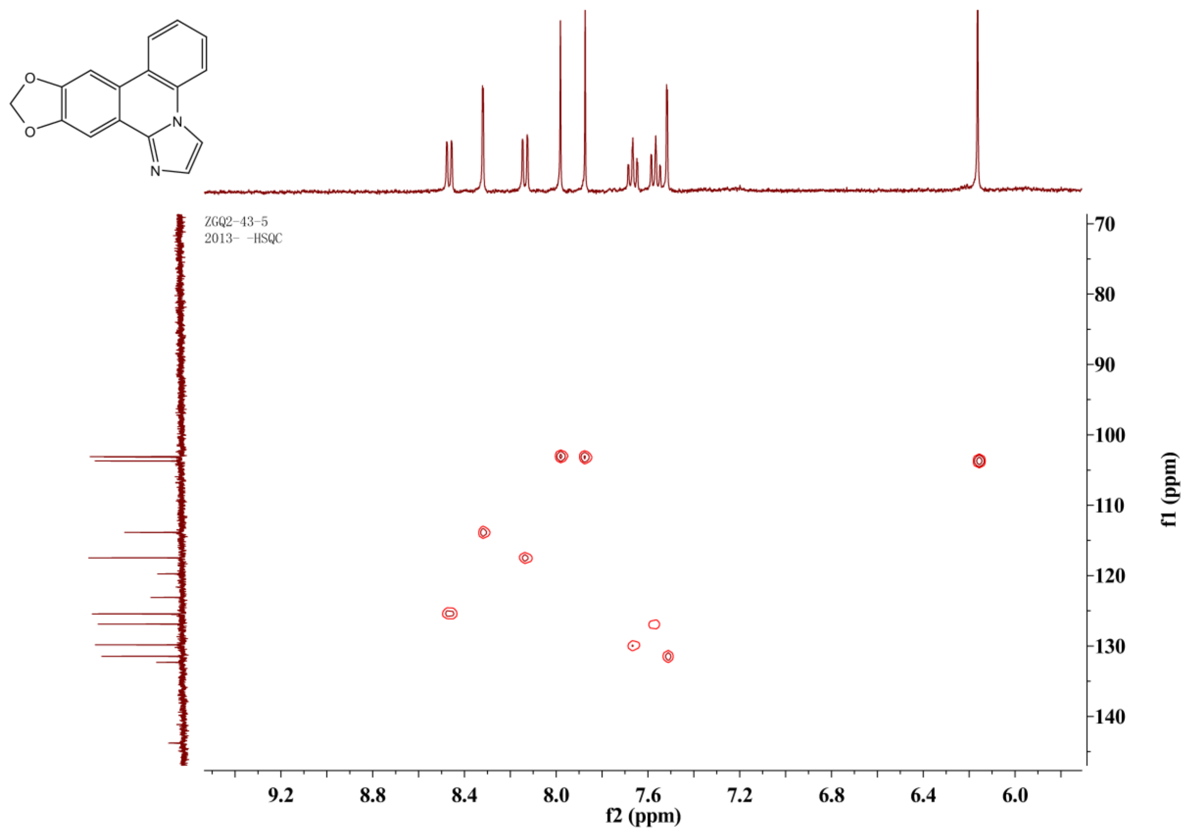


**Figure S13.** HSQC spectrum of **1** ($CD_3OD$)

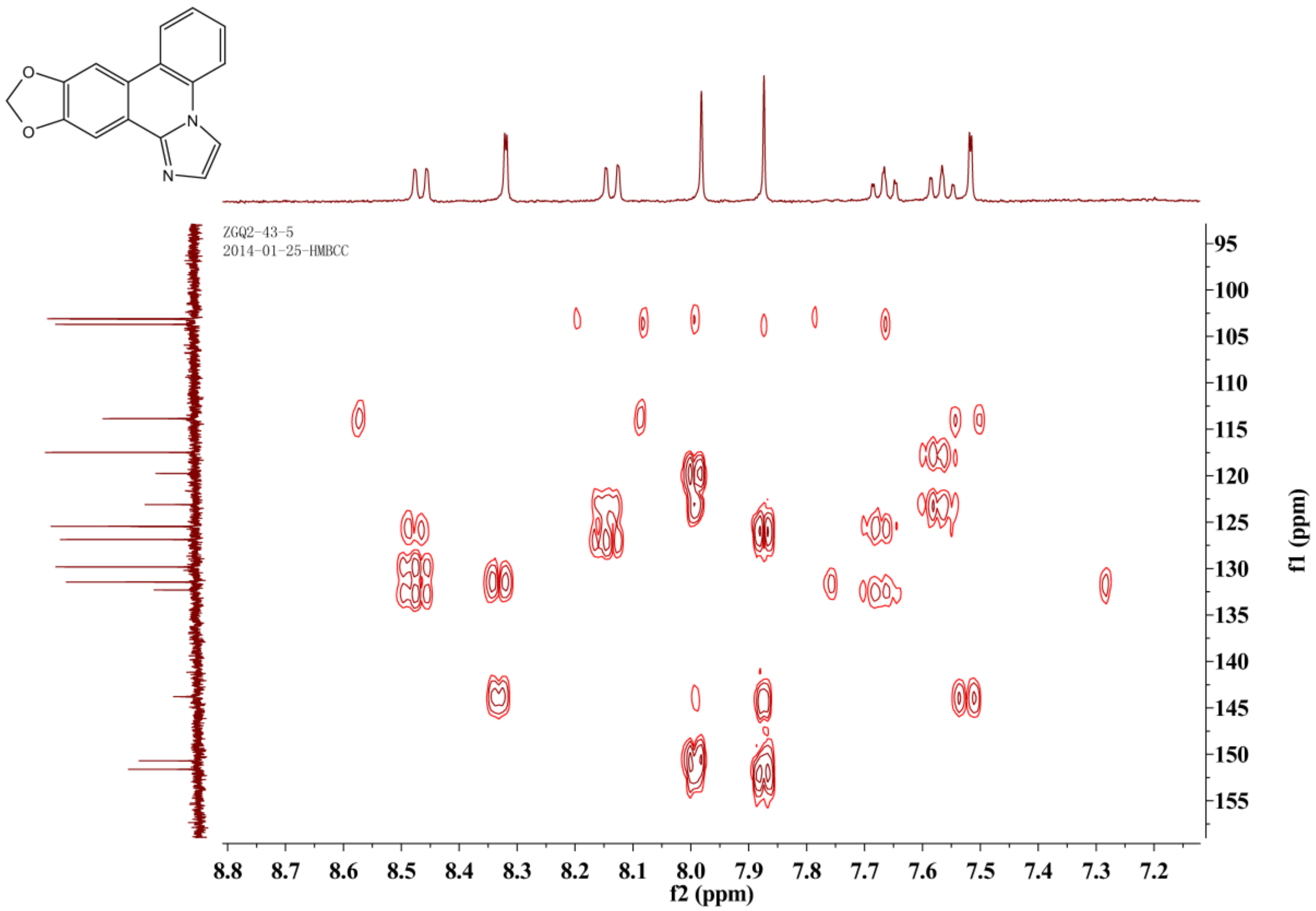


**Figure S14.** HMBC spectrum of **1** ($CD_3OD$)

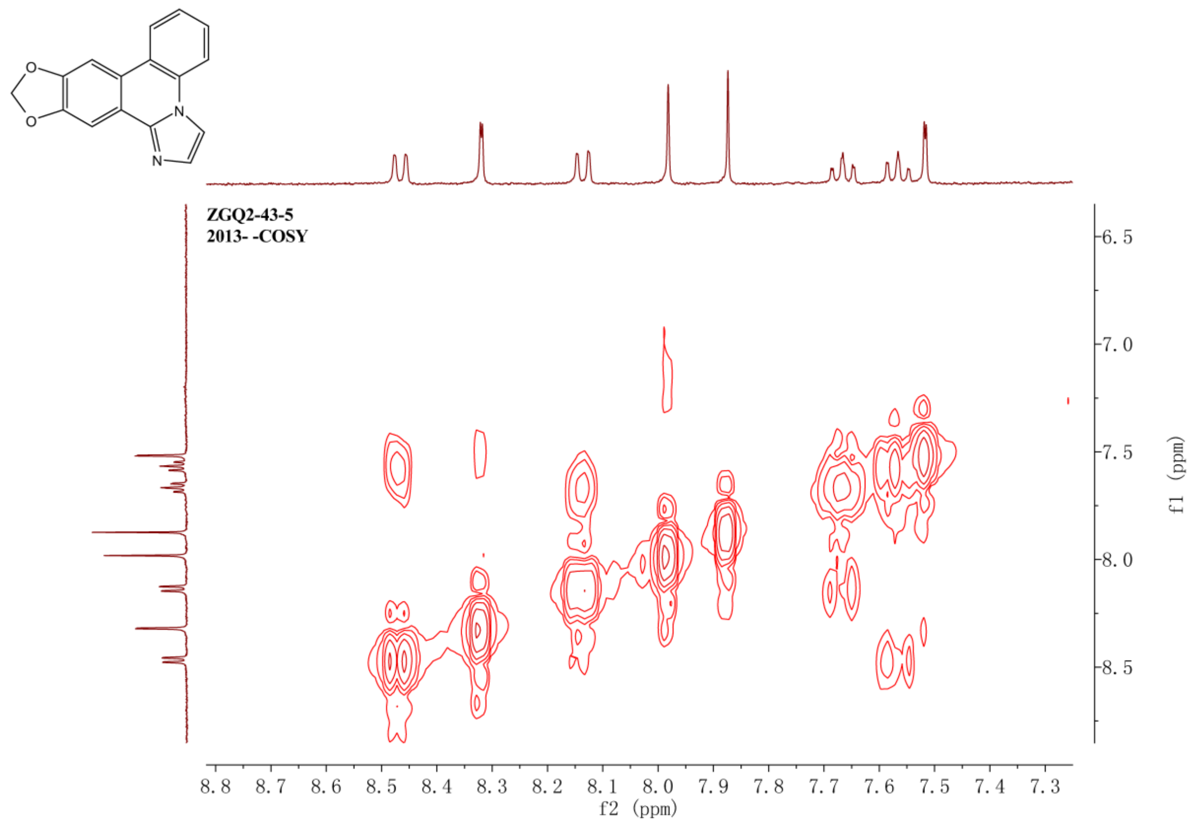


**Figure S15.** $^1H$–$^1H$ COSY spectrum of **1** ($CD_3OD$)

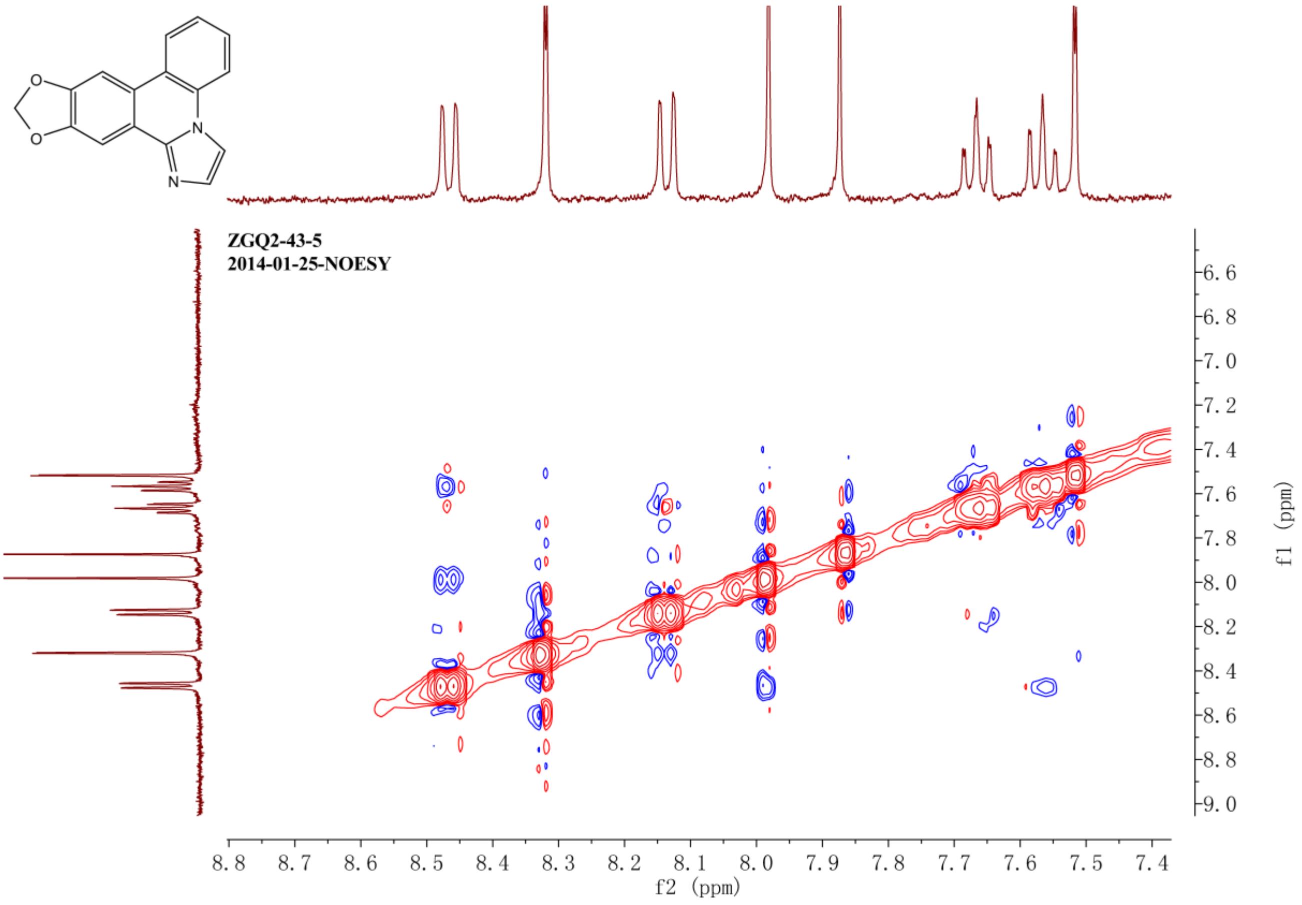


**Figure S16.** NOESY spectrum of **1** ($CD_3OD$)

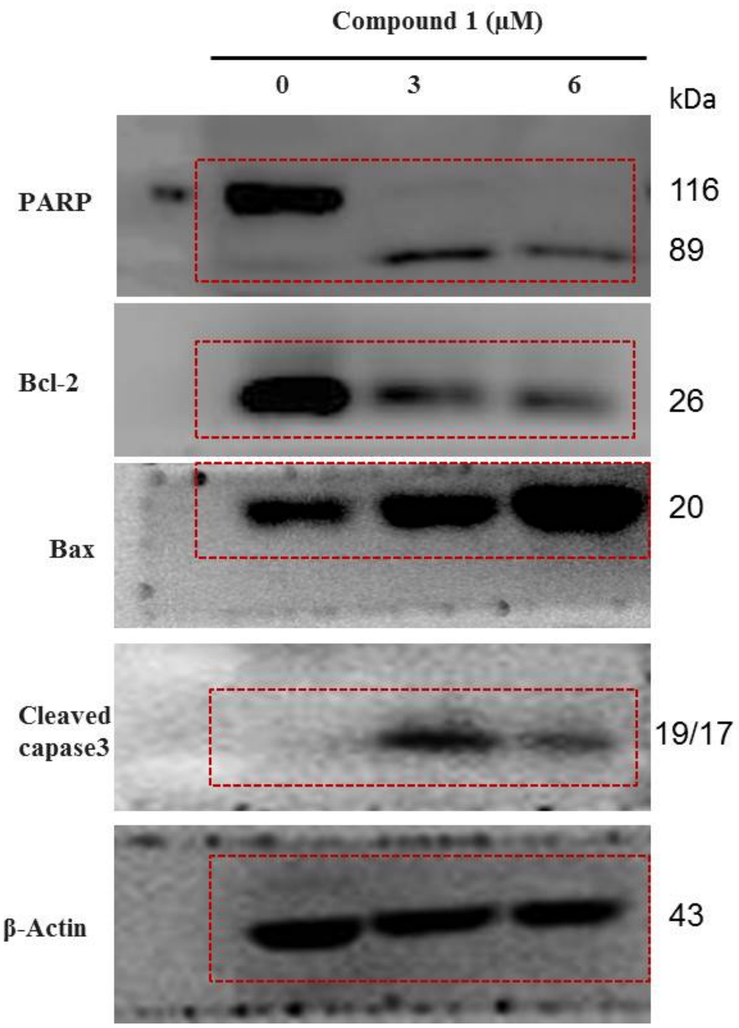


**Figure S17.** Original blots of Figure 5C.

After transfer the blots from the gels to the PVDF membrane, cut the membrane into different parts according the corresponding of the marker and the target proteins. In this case, we could incubate different antibodies with each part at the same time, and then a Bio-imaging System was used for the visualizing of the blots bands. The bands of β-Actin is not in linear is because the membrane was slanted when transferring the blots from the gel to it.
